# The Microbial Flora in an Experimental Polymicrobial Abdominal Sepsis Model Probed by 16S rRNA Sequencing

**Published:** 2020-12-22

**Authors:** Miho Shibamura-Fujiogi, Sophia Koutsogiannaki, Lifei Hou, Koichi Yuki

**Affiliations:** 1 Department of Anesthesiology, Critical Care and Pain Medicine, Cardiac Anesthesia Division, Boston Children's Hospital, USA; 2 Department of Anaesthesia, Harvard Medical School, USA

**Keywords:** Sepsis, Cecal ligation and puncture, 16S rRNA sequencing

## Abstract

**Background:**

Cecal ligation and puncture (CLP) surgery is a widely used preclinical model to induce and study sepsis because it is considered to recapitulate the course of human sepsis the most. This model is highly dependent on the polymicrobial gut flora and represents polymicrobial abdominal sepsis. While the majority of studies using CLP model have focused on the delineation of host immune responses, a limited number of reports have described the composition of microbial strains in this model, although microbial composition can significantly affect the outcome of sepsis in general.

**Methods:**

CLP surgery was performed in mice on C57BL6/J from the Jackson laboratory. We examined the composition of microbes at the peritoneal cavity using 16S rRNA sequencing after CLP surgery at 12 and 24 hours. Baseline cecal microbial flora was also analyzed.

**Results:**

The bacteria strains from the initial cecum flora consisted of mixed aerobic and anaerobic flora. There was a significant change of bacteria flora from the peritoneal cavity between 12 and 24 hours following CLP surgery. Particularly a significantly increased proportion of anaerobic microbes were noted at 24 hours after CLP surgery. We also tested bacterial composition of cecal flora of mice on the same background from the same vendor 6 months later. Baseline cecal microbial flora was different from earlier mice, showing that baseline cecal flora could be different depending on the batch of mice.

**Conclusion:**

There was a dynamical chance of peritoneal microbes during CLP sepsis. Potential difference in baseline cecal flora should be kept in mind upon CLP surgery even when using mice from the same vendor.

## Introduction

Despite a significant improvement of scientific progress and medical care, sepsis remains to be associated with high morbidity and mortality. As the number of sepsis cases has continued to rise, we might have been encountering the increased number of critically ill septic patients as a result of the medical progress. As the diagnosis of sepsis is made when there is a suspected or confirmed infection in the presence of organ dysfunction, it is undoubtedly a quite heterogeneous disease. A single organism may be responsible for sepsis, but polymicrobial infection may be involved. Polymicrobial sepsis is associated with higher morbidity and mortality [1]. For example, colonic perforation and subsequent sepsis carries high morbidity and mortality [2].

The polymicrobial abdominal sepsis model induced by cecal ligation and puncture (CLP) surgery is considered to recapitulate human sepsis most and is the most commonly used sepsis model in rodents [3]. The sepsis pathology in this model is highly dependent on the intrinsic cecal microbial flora. While a number of studies have evaluated host immune responses using this model [3], the investigation of microbial species is surprisingly limited. Because the type of microbes involved in infection is a critical factor to determine the outcome of sepsis, it is important to understand the composition of microbes in the otherwise sterile peritoneal cavity. Microbial culture and subsequent strain identification is a commonly used method to identify microbial species. However, a large number of microbes cannot be cultured in the laboratory setting and this approach will bias the composition of microbes. Here our primary aim is to determine the microbial species at the primary site of infection in this model over time using 16S rRNA sequencing.

## Methods

### Cecal ligation and puncture surgery and sample collection

All the experimental procedures complied with institutional and federal guidelines regarding the use of animals in research. Wild type mice on the C57BL/6J background were purchased from the Jackson Laboratory. Polymicrobial abdominal sepsis was induced by CLP surgery, as we previously performed [4–8]. Briefly, mice were anesthetized with ketamine and xylazine. Following exteriorization, the cecum was ligated at 1.0 cm from its tip and subjected to a single, through and through puncture using an 18-gauge needle. A small amount of fecal material was expelled with gentle pressure to maintain the patency of puncture sites. A part of the fecal material was immediately collected into a tube and stored at −80 °C until use. The cecum was reinserted into the abdominal cavity. 0.1 mL/g of warmed saline was administered subcutaneously. Buprenorphine was given subcutaneously to alleviate postoperative surgical pain. A group of mice were euthanized at either 12 or 24 hours after CLP surgery. Peritoneal lavage fluid was collected and frozen at −80 °C until use. Four mice were assigned to each group.

### 16S rRNA sequencing methods

Samples were thawed on ice and DNA was extracted with the MoBio PowerSoil kit (MoBio Laboratories, Carlsbad, CA) [9]. The V4 hypervariable region of the bacterial 16S rRNA marker gene (16Sv4) was subjected to polymerase chain reaction (PCR) with primers 515F-OH1 (GGACTACNVGGGTWTCTAAT) and 806R-OH (GT-GYCAGCMGCCGCGGTAA) containing unique sequences. The primers contained adaptors for MiSeq sequencing and single-end barcodes allowing pooling and direct sequencing of PCR products. PCR products were subjected to 1% agarose gel to confirm the appropriate size of band. PCR-amplified amplicons were normalized by concentration before pooling and sequences were generated in one lane of a MiSeq instrument using the v2 kit (2 x 250 base paired-end protocol). Sequencing was done at Alkek Center for Metagenomics and Microbiome Research, Baylor College of Medicine (Houston, Texas).

### Bioinformatic analysis

We found total read of 757,083 reads (median 16,849 reads, minimum 6,198 reads, maximum 26,138 reads). 83% (626,717 reads) was mapped and clustered into operational taxonomic units (OTUs) with 97% similarity using QIIME (Version 1.8.1), and taxonomically classified by aligning the representative sequences to the Greengenes 13_08 Database [10]. Alpha and beta diversity and differential abundance analyses were performed in R using the Phyloseq package and DESeq2 as previously described [11].

### Statistical analysis

For each phyla and genera, the within phyla/genera distributions were compared using the two-sample Wilcoxon test with a one-sided alternative as previously performed [12]. P < 0.05 was considered significant.

## Results

### Prolonged isoflurane did not affect the bacterial composition at the peritoneal cavity, but there was a significant difference in post-12 hour and post-24-hour samples

**Figure 1:**
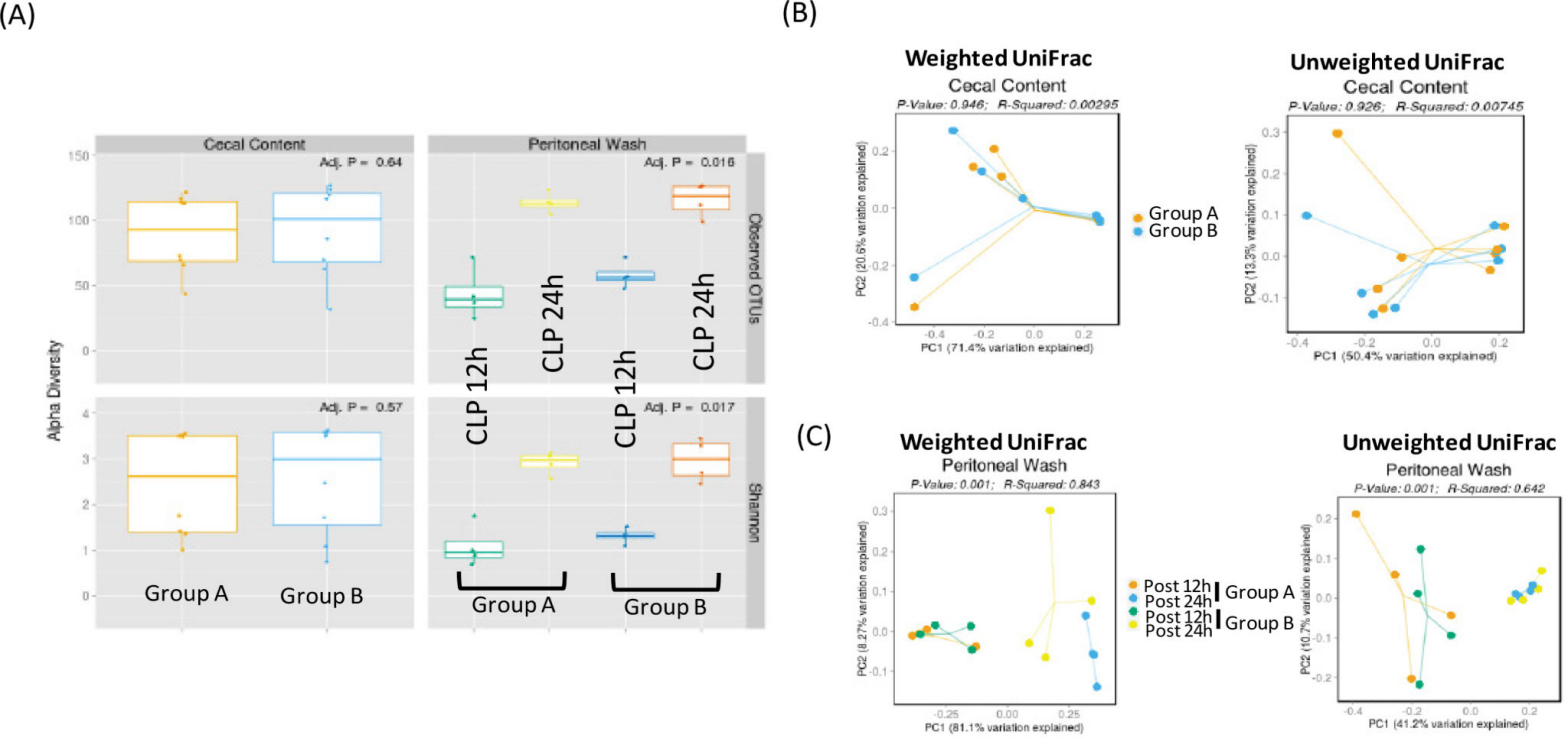
Diversity analysis of cecal and peritoneal space microbes (A) Alpha diversity analysis of cecal and peritoneal space microbes. Observed OTUs and Shannon diversity index were used; (B) Beta diversity analysis of cecal content. Both weighted and unweighted UniFrac were shown; (C) Beta diversity analysis of peritoneal space microbes at two different time points. Both weighted and unweighted UniFrac were shown.

Total of 16 mice were purchased from the vendor at the same time and acclimated for one week in our institution’s animal facility prior to use. 8 mice each were assigned to group A and B. Mice were subjected to CLP surgery. 4 mice of each group were euthanized either at 12 hours or 24 hours after CLP surgery for peritoneal bacterial analysis. Alpha diversity is used to characterize the richness of microbes and their heterogeneity within samples. Based on the number of observed OTUs, there was no significant difference in the number of -mi crobes at the baseline cecum content between Group A and Group B, suggesting that there was no significant difference in the composition of microbes spilled into the peritoneal cavity by CLP surgery between the two groups (Figure 1A). Shannon diversity index is another index that is commonly used to characterize species diversity. There was also no difference in Shannon diversity index of cecal microbes between Group A and Group B (Figure 1A). Beta-diversity is used to characterize diversity between samples. UniFrac is phylogenetic-based beta-diversity. When taxonomic abundance is weighed into a consideration, it is called weighted UniFrac, where dominant microbes become more prominent. When just focusing on dissimilarity based only on phylogenetic difference without any consideration of microbial abundance, it is called unweighted UniFrac. In this case, less abundant microbes can be noted more. The comparison of cecal microbes at the time of CLP did not show any difference in beta diversity (Figure 1B). These data confirmed that the baseline cecal microbes of the two groups were not different.

At both 12 and 24 hours, there was no difference of microbial population in the peritoneal cavity between Group A and Group B based on alpha diversity (Figure 1A). Instead there was a significant difference in microbial diversity between post 12-hour and post 24-hour samples based on the beta diversity analysis (Figure 1C).

### The proportion of anaerobic microbial species increased at 24 hours after CLP surgery

**Figure 2:**
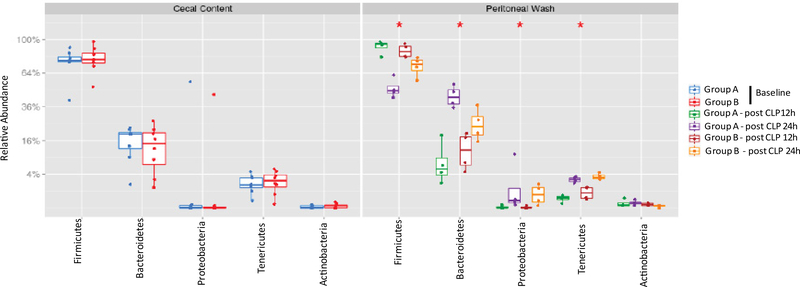
The analysis of most abundant phyla in cecal content and peritoneal space. Relative abundance of five most abundant phyla was shown. Statistical analysis was performed using Wilcoxon test within the phyla. No statistical significance was observed between Group A and Group B.

Five most abundant phyla were Firmicutes, Bacteroidetes, Proteobacteria, Tenericutes and Actinobacteria in the cecal content as well as in the peritoneal cavity after CLP surgery (Figure 2). Between post 12-hour and post 24-hour samples, we observed a decreased proportion of Firmicutes and an increased proportion of Bacteroidetes, Proteobacteria and Teneticutes.

**Figure 3:**
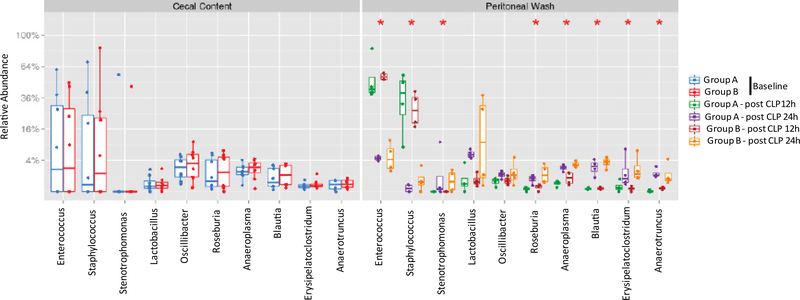
The analysis of most abundant genera in cecal content and peritoneal space. Relative abundance of ten most abundant genera was shown. Statistical analysis was performed using Wilcox test within the genera comparing two time points or between the groups. No statistical significance was identified between the two groups at the same time point, but statistical significance was observed between 12-hour and 24-hour samples. *p < 0.05

Next, we examined the top 10 abundant genera. *Enterococcus*, *Staphylococcus*, *Sternotrophomonas*, *Lactobacillus*, *Oscillibacter*, *Roseburia*,*Anaeroplasma*, *Blautia*, *Erysipelatoclostridium* and *Anaerotruncus* were among the top 10 genera. From 12 hours to 24 hours after CLP, a significantly decreased proportion of *Enterococcus* and *Staphylococcus* was observed. Simultaneously a significantly increased proportion of *Sternotrophomonas*, *Roseburia*, *Anaeroplasma*, *Blautia*, *Erysie-patoclostridium* and *Anaerotruncus* were observed (Figure 3). There was a tendency of increased proportion of Lactobacillus, although not statistically significant. *Enterococcus* and *Staphylococcus* were aerobic in nature, while the rest of microbes were anaerobic. There was no difference in the proportion of microbial composition between Group A and Group B.

**Figure 4:**
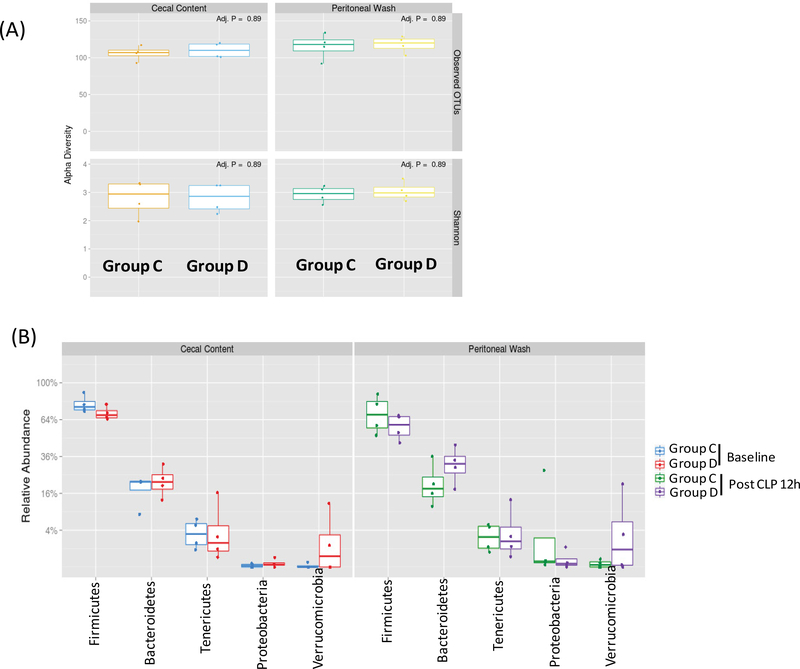
The analysis of microbial diversity and most abundant phyla in cecal content and peritoneal space (A) Alpha diversity analysis of cecal and peritoneal space microbes. Observed OTUs and Shannon diversity index were used; (B) Relative abundance of five most abundant phyla was shown. Statistical analysis between the groups was performed using Wilcoxon test within the phyla. No statistical significance was observed.

**Figure 5:**
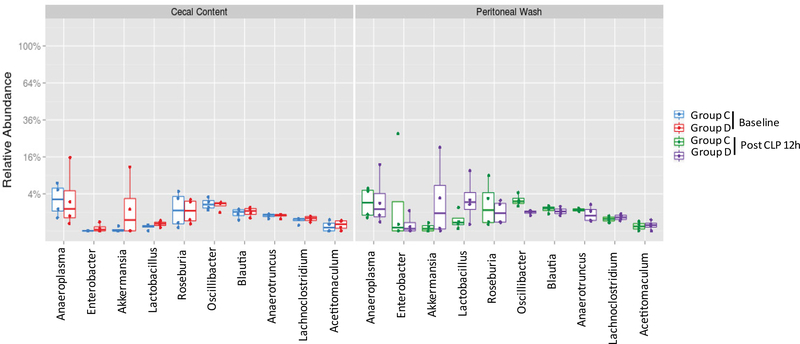
The analysis of most abundant genera in cecal content and peritoneal space. Relative abundance of ten most abundant genera was shown. Statistical analysis was performed using Wilcox test between the groups.

In a separate experiment, we also obtained cecal content from 8 wild type mice that were purchased from the same vendor in 6-month apart and acclimated for one week before CLP surgery. 4 mice each were divided into Group C and Group D. There was no difference between cecal content and peritoneal bacteria flora (Figure 4A). Top 5 most abundant phyla were Firmicutes, Bacteroidetes, Tenericutes, Proteobacteria, and Verrucimicrobia (Figure 4B). Top 10 genera were *Anaeroplasma*, *Enterobacter*, *Akkermansia*, *Lactobacillus, Roseburia*, *OScillibacter*, *Blautia*, *Anaerotrunctus*, *Lachnoclostridium* and *Acetitomaculum* (Figure 5), suggesting that baseline cecal flora characteristics could be different between two separate experiments.

## Discussion

Here we showed that 1) The proportion of microbial composition in the peritoneal cavity changed following CLP surgery, with an increased proportion of anaerobic microbes at 24 hours after CLP surgery, 2) Baseline cecal microbial flora could be quite different between a batch of micefrom the same vendor.

The metagenomic analysis has been increasingly used to understand microbiome. Historically, identification of microbial members is culture-dependent. Once they grow, they are often subjected to staining such as Gram stain, morphological analysis and growth on different media [13]. However, the vast majorities of microbial species have never been grown in the laboratory. Hyde, et al. examined the microbial population by culturing blood and other organs at 2 and 12 hours after CLP and at the time of death under both aerobic and anaerobic conditions [14]. Blood culture predominantly showed anaerobes at 2 hours, but aerobes became a major population at the time of death. Given that blood is highly oxygenated, this change is intuitive. Some of anaerobic bacterial infection is associated with a significantly high mortality, and it is also interesting to understand the role of individual anaerobes in sepsis outcome. The result of microbial composition in the peritoneal cavity in our study is in clear contrast to the blood culture results by Hyde, et al. Performing 16S rRNA seq in blood samples can be also considered in the future.

We noted the baseline microbial flora of mice could be quite different even purchased from the same vendor and housed in the same facility. This difference may affect the phenotype of sepsis and be potentially problematic. Previously, Fay, et al. examined the microbiota of C57/B6 mice from the two different vendors (Jackson laboratory, Charles river) and also performed CLP surgery soon after receiving them [15]. They found that the mice from Jackson laboratory had less α-diversity with worse survival and different T cell profiles. However, after co-housing them for three weeks, the survival following CLP surgery did not show any difference with similar T cell profiles, indicating that the microbiota is highly responsible for immune phenotype as well as sepsis outcome. In human case reports, a different microbiome-based therapy and fecal microbiota transplantation have shown that restoration of the gut microbiota along with decreased inflammatory responses [16], indicating human relevance of the findings. A cecal slurry peritoneal injection was proposed to be an alternative model for sepsis to obtain consistent sepsis phenotype [17]. In this model, cecal content is collected as a batch and stocked for the future injection, which allows mice to receive the same composition of polymicrobes. The cecum is ligated for CLP surgery, which could create ischemic tissue. In addition, it is unclear if all the microbes stocked for a cecal slurry would be viable at the time of injection. Thus, CLP sepsis and cecal slurry peritoneal injection sepsis model may not be necessarily the same, but complementary approach may be useful [18].

In our study, we focused on the microbial flora at the peritoneal cavity, the original source of infection. pH, oxygen level, nutrient supply, and redox potential serve as strong selective pressures for particular microbes. Furthermore, chemical interactions through interspecies signals and metabolites also affect the composition of microbes in polymicrobial condition [19]. The major limitation of the study is that we did not perform pH, nutrient supply and redox potential in the peritoneal cavity due to the technical difficulty. Our study suggested that the proportion of anaerobic strains increased over time in the peritoneal cavity. Because microbial cohabitation is such a complicated process, studying the underlying mechanism of this transition remains to be done in the future.

In conclusion, we have shown that the decreased proportion of aerobic strains and the increased proportion of anaerobic strains were observed in the peritoneal cavity at a later time point after CLP surgery. We also showed that bacterial flora can be different depending on the batch of mice.

## Abbreviation

CLP: Cecal ligation and puncture
PCR: Polymerase chain reaction
